# Autoantibodies and gastrointestinal symptoms in infertile women in relation to in vitro fertilization

**DOI:** 10.1186/1471-2393-13-201

**Published:** 2013-11-05

**Authors:** Oskar Hammar, Bodil Roth, Mariette Bengtsson, Thomas Mandl, Bodil Ohlsson

**Affiliations:** 1 Department of Clinical Sciences, Division of Internal Medicine, Skåne University Hospital, Lund University, 205 02, Malmö, Sweden; 2 Faculty of Health and Society, Institution of Care Science, Malmö University, Malmö, Sweden; 3 Department of Clinical Sciences, Division of Rheumatology, Skåne University Hospital, Lund University, 205 02, Malmö, Sweden

**Keywords:** Abdominal pain, Gastrointestinal symptoms, Gonadotropin-releasing hormone, In vitro fertilization, Luteinizing hormone

## Abstract

**Background:**

Prior reports suggest a link between gonadotropin-releasing hormone (GnRH) and gastrointestinal function. The aim of the study was to prospectively investigate women subjected to in vitro fertilization (IVF) using the GnRH analog buserelin, taking into account gastrointestinal symptoms and antibody development against buserelin, GnRH, luteinizing hormone (LH), and their receptors.

**Methods:**

Gastrointestinal symptoms were registered by the Visual Analogue Scale for Irritable Bowel Syndrome (VAS-IBS) before and after IVF treatment, and five years later. Health-related quality of life was evaluated by the 36-item Short-Form questionnaire (SF-36). ELISA was used for antibody analyses before and after treatment. Data were compared with women from the general population.

**Results:**

In total, 124 patients were investigated before and after IVF, and 62 were re-evaluated after five years. Buserelin treatment led to significant impairment of constipation (p = 0.004), nausea and vomiting (p = 0.035), psychological well-being (p = 0.000), and the intestinal symptoms’ influence on daily life (p = 0.027). At 5-year follow-up, abdominal pain was worsened (p = 0.041), but psychological well-being was improved (p = 0.036), compared to prior treatment, and 15% had an observable deterioration in gastrointestinal symptoms. None developed severe dysmotility. Patients had higher prevalence of IgG antibodies against LH (p = 0.001) and its receptor (p = 0.016), and IgM antibodies against the GnRH receptor (p = 0.001) prior treatment compared with controls, but no antibody development was observed after IVF.

**Conclusion:**

Patients experience gastrointestinal symptoms during buserelin treatment, and abdominal pain is still increased after five years, but buserelin does not increase antibody formation against GnRH, LH or their receptors.

## Background

Irritable bowel syndrome (IBS) affects approximately 10%–15% of the western population, women 1.5–3 times more often than men [1]. To explain why women are affected to a larger extent than men, connections between sex hormones, particularly progesterone, and gastrointestinal function have been proposed [2,3]. Gonadotropin-releasing hormone (GnRH) is the hypothalamic hormone in the sex hormone axis, which stimulates release of follicle-stimulating hormone (FSH) and luteinizing hormone (LH), and subsequently estrogen and progesterone [4].

The role of GnRH in gastrointestinal function has only been rudimentarily examined. Huang W et al. [5] have shown GnRH- and GnRH receptor (GnRH-R) immunoreactivity in the epithelium and myenteric ganglia of small and large rat intestine, and the GnRH analog leuprolide acetate has by unknown mechanisms been shown to stimulate cycling motor activity in rat gut [6]. Gonadotropin-releasing hormone is also present in the human enteric nervous system (ENS) [7], and antibodies against the peptide are more common in IBS- and dysmotility patients as compared with controls [8]. Continuous treatment with leuprolide significantly decreased nausea, abdominal pain, early satiety, anorexia, and abdominal distension in patients with functional bowel disease [9,10]. On the contrary, chronic intestinal pseudo-obstruction (CIPO) was developed after repeated treatment with the GnRH analog buserelin in the setting of in vitro fertilization (IVF). Full-thickness biopsy showed enteric neurodegeneration with almost total absence of GnRH-containing neurons [11]. Scrutiny of 22 patients who had undergone full-thickness biopsy due to severe, gastrointestinal motility disorders, i.e. CIPO or enteric dysmotility (ED), revealed five patients with lowered levels of enteric GnRH-containing neurons and elevated levels of serum antibodies against GnRH. Three of these five had had repeated treatments with GnRH analogs in an IVF setting and/or due to endometriosis [7]. Repeated buserelin treatment of rats led to 50% loss of enteric neurons [12].

As IVF is given repeatedly to young women, the same population which is most likely to be affected by gastrointestinal symptoms and dysmotility [1], and sporadic cases of severe dysmotility have been reported after IVF [7,11], we found it important to examine possible connections between IVF and IBS or dysmotility in an IVF cohort. The aim of the present study was thus to prospectively investigate women subjected to IVF using buserelin treatment, taking into account gastrointestinal symptoms in relation to treatment as well as five years later. The presence of antibodies against buserelin, GnRH, LH, and their receptors, before and after treatment, were also evaluated.

## Methods

This study was approved by the Ethics Review Board of Lund University and performed in accordance with the declaration of Helsinki. All subjects gave their written, informed consent before inclusion in the study. Naïve blood samples from the patients taken during the very first pre-IVF screening was used in accordance with the Swedish Act ’Biobanks in Medical Care Act‘ (SFS 2002:297).

### Patients

Patients were recruited to the study at a fertility clinic in Malmö where they sought care for infertility. The clinic receives patients from the southernmost districts of Sweden. The reasons for the infertility were not further investigated. After appropriate consultation, an IVF regime was planned. One selected nurse was responsible for recruiting consecutive patients passing her with a planned regime involving the GnRH analog buserelin, from 2007 through 2008.

### Controls

Two age- and gender-matched controls for each included patient were randomly acquired from the Swedish Population Registry at the 5-year follow-up. Thus, 248 women were contacted via mail. After one reminder, 29 questionnaires were returned. As the response rate was low, further controls were recruited amongst hospital staff. In total, 65 healthy women, median age 37 (IQR 34–42) years, were recruited and completed the questionnaires.

Blood samples from 169 consecutive healthy female blood donors, median age 45 (IQR 31–54) years, collected at Skåne University Hospital, Malmö, served as a control group for antibody analyses within the study.

### Study design

**Figure 1:**
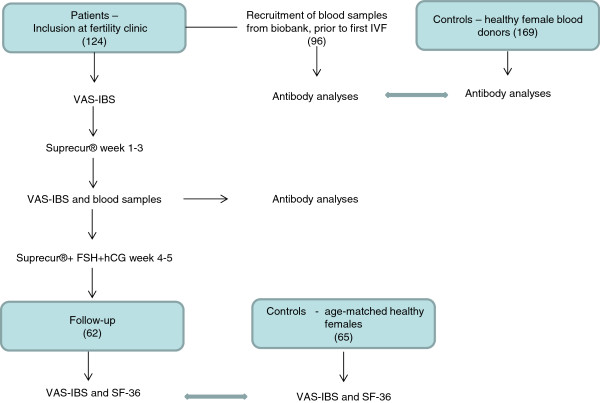
**Flow chart over the study design.** VAS-IBS = Visual Analogue Scale for Irritable Bowel Syndrome. FSH = Follicle-stimulating hormone, hCG = human chorionic gonadotropin, IVF = In vitro fertilization.

Figure 1 illustrates the study design. Patients underwent IVF according to clinical routines. The Visual Analogue Scale for Irritable Bowel Syndrome (VAS-IBS) was completed and the IVF treatment started with two nasal inhalations of the GnRH analog buserelin (Suprecur®, Sanofi-Aventis, Bromma, Sweden) four times a day for three weeks; dose was varying according to clinical response. The VAS-IBS was again completed after these three weeks and blood samples collected. Blood samples consisted of 7.0 ml whole blood drawn into heparinized or untreated tubes. Plasma and serum were separated and immediately frozen at −20°C. This was followed by one inhalation four times a day for another two weeks, along with FSH and human chorionic gonadotropin (hCG) administered according to clinical routines. The total dose of buserelin was 28.8 (IQR 27.0–31.6) mg. Treatment naïve blood samples were obtained from the Department of Medical Microbiology, Skåne University Hospital, Malmö. Sera were analyzed for the presence of antibodies against buserelin, GnRH, LH, and their receptors.

Five years after the initial treatment, the VAS-IBS questionnaire and the 36-item Short-Form questionnaire (SF-36) were sent to patients and matched controls. One reminder was sent within one month, if no reply was returned.

### Questionnaires

#### Visual Analogue Scale for Irritable Bowel Syndrome

The VAS-IBS was used to estimate gastrointestinal complaints. This is a validated questionnaire for estimation of the most common gastrointestinal complaints in patients with functional, non-organic bowel disease [13]. This instrument has also been validated for evaluation of symptoms over time [14]. The seven VAS items used assess each of the following symptoms: abdominal pain, diarrhea, constipation, bloating, nausea and vomiting, psychological well-being, and intestinal symptoms’ influence on daily life. The scale measures from 0–100 mm, where 0 represents very severe problems and 100 represent absence of problems. Two questions at the end of the form are to be answered with yes or no; feeling of incomplete evacuation and presence of tenesms.

#### 36-item Short-Form questionnaire

The SF-36 was used to assess health-related quality of life (HRQOL). It is a generic questionnaire divided into eight subscales of general health and ordered according to which degree they measure physical or mental health. Four subscales correspond mainly to the Physical Composite Score (PCS), namely, physical functioning (PF), role physical (RP), bodily pain (BP), and general health (GH). Four subscales correspond mainly to the Mental Composite Score (MCS), namely, vitality (VT), social functioning (SF), role emotional (RE), and mental health (MH). The subscales GH, VT, and SF also correspond with the PCS [15]. SF-36 is an extensively used HRQOL instrument, which provides reproducible, reliable data in large populations and is used as a global health monitor [16]. The maximum score is 100, the higher score the better the HRQOL. Norm-based scores for the SF-36 questionnaire were calculated for patients as well as for controls, using the QualityMetric Health Outcomes™ Scoring Software 4.5 [17].

#### Measurement of antibodies against buserelin, GnRH and GnRH receptor

Analyses of antibodies against GnRH and GnRH-R were carried out by an ELISA as described previously [18]. The wells of microtiter plates (456537 Nunc, Roskilde, Denmark) were either coated with buserelin (Suprefact® lot 2 F0174, Sanofi-Aventis, Bromma, Stockholm), or human GnRH or N-terminal GnRH-R peptide ((NH2)-ANSASPEQNQNHCSAINNSIPLMQGNLPY) conjugated with ovalbumin (OVA) (Innovagen, Lund, Sweden), in an overnight incubation at 4°C. Thereafter the plastic wells were blocked with 0.5% bovine serum albumin (BSA) (A-7030, Sigma, St Louis, USA) in phosphate-buffered saline (PBS) containing 0.05% Tween-20 (PBS-T). The dilutions of patient serum (1:400 in 1.0 μg OVA (A-5503, Sigma)/ml 0.5% BSA in PBS-T) or mouse anti-human GnRH antibody (ab62432, Abcam, Cambridge, MA, USA) in serial dilution (to construct a standard curve) were then added to the plates and incubated for 2 h at room temperature (RT). After rinsing with PBS-T, deposition of antibodies directed to buserelin, GnRH or GnRH-R was detected using biotinylated rabbit anti-human IgM- (673211, MP Biomedicals, Solon, OH, USA), IgG- (ab7159, Abcam), or IgA antibodies (ab97218, Abcam), or goat anti-mouse IgG antibodies (E0433, DAKO, Glostrup, Denmark) appropriately diluted in PBS-T. After another incubation for 2 h at RT, the plates were washed and the bound, biotinylated antibodies detected by alkaline phosphatase-conjugated streptavidin (405211, Biolegend, San Diego, CA, USA), incubated for 1 h at RT. To develop a color reaction, a phosphatase substrate kit (37620, Pierce, Rockford, Ill, USA) was used. The absorbance at 405 nm was measured after 30 min of incubation at RT. Antibody levels are expressed as relative units (RU) (absorbance values after subtracted background levels and multiplied with 1000). The cut-off value to determine presence of antibodies in the control group of 169 healthy female blood donors was defined as RU >97.5^th^ percentile. The intra-assay correlation coefficient of variation (CV) of GnRH- and GnRH-R IgM antibodies was 10% and 8%, respectively (n = 6), and inter-assay CV was 11% and 6%, respectively (n = 12). No intra-assay or inter-assay CV of IgG antibodies, IgA antibodies or buserelin antibodies was calculated due to lack of positive serum.

For competitive ELISA, diluted sera from patients with antibodies above the cut-off level were incubated in 0.05% BSA in PBS-T with various amounts of GnRH or GnRH-R peptide, both unconjugated and conjugated with OVA (Innovagen), prior to application to the microtiter plate.

#### Measurement of antibodies against LH and LH receptor

An in-house ELISA was set up for analysis of anti-IgG- and anti-IgM antibodies against LH and its receptor (LH-R).

#### LH

The microtiter plates (456537, Nunc) were coated with intact, purified, native human LH (MBS537383, MyBiosource, San Diego, CA, USA) in PBS-T or only PBS-T (to provide an internal blank). After overnight incubation at 4°C, the plates were washed three times with PBS-T and blocked with 0.5% BSA in PBS-T. Dilutions of serum (1:200) from patients and blood donors, or rabbit anti-human LH antibody (MBS535386, MyBiosource) in serial dilution (to construct a standard curve), with BSA in PBS-T were then added to the plates in triplicate (two wells coated with LH and one well coated with PBS-T) and incubated for 2 h at RT. The washing procedure was repeated and deposition of autoantibodies directed to LH was detected using biotinylated rabbit anti-human IgM- (673211, MP Biomedicals) or IgG antibodies (ab7159, ABcam), or goat anti-rabbit IgG antibodies (B7389, Sigma) appropriately diluted in PBS-T.

#### LH receptor

Microtiter plates were coated with the N-terminal LH-R peptide ((NH2)-MKQRFSSALQLLKLLLLQPPLPRALC), conjugated with OVA (Innovagen), in 100 mM Carbonate buffer pH 9.2 or only Carbonate buffer (to provide an internal blank). After overnight incubation at 4°C, the plates were washed three times with PBS-T and blocked with 0.5% BSA in PBS-T. Dilutions of serum (1:200) from patients and blood donors with BSA in PBS-T were then added to the plates in triplicate, two to LH-R and one to Carbonate buffer-coated wells, and incubated for 2 h at RT. The washing procedure was repeated and deposition of autoantibodies directed to LH-R was detected using biotinylated rabbit anti-human IgM- (673211, MP Biomedicals) or IgG antibodies (ab7159, Abcam) appropriately diluted in PBS-T.

To develop a color reaction, a phosphatase substrate kit (37620, Pierce) was used. The absorbance at 405 nm was measured after 30 min (LH IgM and LH-R) or 60 min (LH IgG) of incubation at RT. Antibody levels are presented as RU (absorbance values after subtracted background levels and multiplied with 1000) and the cut-off value to determine presence of antibodies in the control group of 169 healthy female blood donors was defined as RU >97.5^th^ percentile. The intra-assay CV of LH IgG and LH IgM was 5.6% and 9.2%, respectively (n = 8), and inter-assay CV of LH IgG and LH IgM was 7.7% and 6.1%, respectively (n = 17). No intra-assay or inter-assay CV of LH-R was calculated due to lack of appropriate commercial antibody.

For competitive ELISA, diluted sera from patients with antibodies above the cut-off level were incubated in 0.5% BSA in PBS-T with various amounts of LH (MBS537383, MyBiosource) or LH-R, unconjugated and conjugated with OVA (Innovagen), i.e. 50, 100, or 200 ng/100 μl, 30 min prior to application to the microtiter plates.

#### Statistical methods

The data were analyzed using the statistical software package SPSS for Windows© (Release 20.0; IBM). All variables were analyzed for normal distribution by Kolmogorov-Smirnov test. As normality was rejected, the Wilcoxon’s signed rank test was used to calculate differences within the group and the Mann-Whitney U-test to compare different groups. When appropriate, Fisher’s exact test was used. Spearman’s rank correlation test was applied for correlations. Values are expressed as median, interquartile range (IQR). P < 0.05 was considered statistically significant.

## Results

### Patient characteristics

The median age of the 124 included patients was 34 years, IQR 31–37 years, at the time of IVF treatment. All except one completed the VAS-IBS questionnaire. Twelve percent were current smokers. An anamnesis of past or concurrent diseases was present in 51 patients (49%). The most common diseases were endometriosis (15 (12%)), asthma (8 (6%)), and hypothyroidism (8 (6%)) (Additional file 1: Table S1). Thirty-two percent of the patients reported some kind of allergy. Only one of the patients was currently unemployed. Forty percent of the women were subjected to IVF treatment for the first time, 24% had IVF treatment for the second time, 23% for the third time, and 14% had had four or more treatments when investigated. At most, one patient had had eight prior IVF treatments.

### Gastrointestinal symptoms before and after buserelin treatment

The patients had more nausea and vomiting already before treatment compared with controls (95 (87–98) and 98 (92–99), respectively, p = 0.011).

Comparing VAS-IBS before and after treatment, buserelin had significant negative effects on constipation, nausea and vomiting, psychological well-being, and the intestinal symptoms’ influence on daily life (Table 1). The increased abdominal pain and bloating after treatment showed a tendency towards significance, p = 0.052 and p = 0.079, respectively, whereas diarrhea was not significantly influenced by the treatment (Table 1). No correlation was found between age and VAS-IBS parameters (data not shown). No differences were detected regarding feeling of incomplete evacuation and need to defecate (data not shown).

**Table 1:** VAS-IBS questionnaire scores before and after in vitro fertilization

	**Before N = 123**	**After N = 123**	**Follow-up N = 62**	**Controls N = 65**	**Before vs.**	**Before vs.**	**Before vs.**
	**median (IQR)**	**median (IQR)**	**median (IQR)**	**median (IQR)**	**After**	**Follow-up**	**Controls**
Abdominal pain	92 (79–97)	86 (68–96)	84 (71–97)	93 (73–98)	0.052	0.041	0.881
Diarrhea	94 (82–97)	93 (80–96)	95 (78–98)	95 (80–98)	0.617	0.778	0.812
Constipation	93 (79–97)	86 (67–96)	93 (73–97)	95 (83–98)	0.004	0.839	0.107
Bloating	77 (61–94)	74 (50–88)	73 (59–95)	80 (39–95)	0.079	0.850	0.861
Nausea and vomiting	95 (87–98)	95 (84–97)	97 (93–99)	98 (92–99)	0.035	0.305	0.011
Psychological well-being	87 (69–96)	78 (52–93)	93 (79–98)	85 (74–96)	0.000	0.036	0.682
Intestinal symptoms’ influence on daily life	93 (77–98)	90 (77–96)	94 (70–98)	96 (75–98)	0.027	0.928	0.703

A lower value of the Visual Analogue Scale for Irritable Bowel Syndrome (VAS-IBS) indicates worse symptoms. Values are given as median, interquartile range (IQR). Wilcoxon’s signed rank test or Mann-Whitney U-test were used. P < 0.05 was considered statistically significant.

### 5-year follow-up of gastrointestinal symptoms and health-related quality of life

Sixty-two of the initial 124 included women (49%) returned their questionnaires at follow-up after one reminder. There was no difference in base-line characteristics or VAS-IBS scores between those who returned their questionnaires after five years or not (data not shown). Abdominal pain had been worsened at the 5-year follow-up, but psychological well-being had been improved as compared with the measurements before treatment (Table 1). Nine of 62 patients (15%) had prominent negative deviations in VAS-IBS compared with before treatment. These were contacted by telephone, and the aggravation in symptoms seemed to be explained by development or exacerbations of IBS symptoms. Three of these had become pregnant and got children after IVF, and two of them had an anamnesis of endometriosis. None had developed severe dysmotility. Two patients had prominent positive deviations in VAS-IBS at follow-up. There was no difference in VAS-IBS between controls and patients at 5-year follow-up (data not shown). There was no difference in abdominal pain between those patients with an anamnesis of endometriosis or not, at any time point of completion of the questionnaire (p = 0.341, p = 0.312, and p = 0.114, respectively).

**Figure 2:**
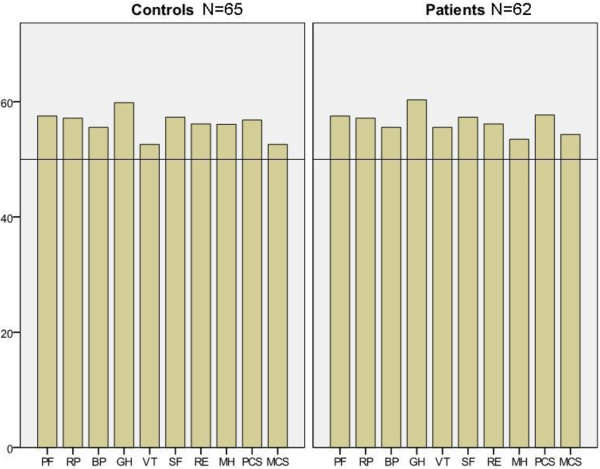
**Self-reported health-related quality of life using the Short Form 36 (SF-36) questionnaire in 65 controls and 62 patients.** Four subscales correspond mainly to the Physical Composite Score (PCS): PF = Physical functioning, RP = Role physical, BP = Bodily pain and GH = General health. Four subscales correspond mainly to the Mental Composite Score (MCS): VT = Vitality, SF = Social functioning, RE = Role emotional, MH = Mental health. Subscales GH, VT, and SF also correspond with the PCS. Median values of norm-based scores for the different groups are presented. The Quality metric software employs a linear T-score transformation with mean = 50 and standard deviation = 10, utilizing normative data from the QualityMetric 2009 General Population Sample [17].

Norm-based scores from SF-36 were above norm values for all domains in controls and patients five years after treatment, using normative data from the QualityMetric 2009 general population sample (Figure 2). The only significant difference across groups was in the subscale role emotional, where patients scored norm-based median 56.2 (55.3–56.2) compared to 56.2 (49.2–56.2) in controls (p = 0.012). No correlations could be found between the number of IVF treatments and any of the VAS-IBS- or SF-36 variables (data not shown).

### Antibody measurements

All antibodies measured were found in low prevalence in controls, without any age-related differences in prevalence. At most, 1–3 subjects in every 10-year period expressed antibodies. Sera were available both before and after IVF treatment in 96 of the 124 patients (77%). IgA- and IgG antibodies against GnRH and GnRH-R were uncommon in both patients and blood donors, and were not further analyzed since none or maximum two individuals were regarded as positive in each group. No antibodies against buserelin were detected. Regarding IgM antibodies against GnRH and GnRH-R, patients expressed GnRH-R IgM antibodies to a higher extent before treatment as compared with controls (Table 2). Competitive ELISA showed that the antibody-binding capacity to the solid phase was reduced with 25%–63%, dependent on the amount of antigen added (1.0–5.0 ng/ml) to the diluted serum before application on the microtiter plate. There was no difference before or after buserelin treatment in GnRH- and GnRH-R antibody prevalence or level (Tables 2 and 3). Two patients had a prominent increase (about 100 fold) in GnRH-R antibody concentrations during IVF treatment. One of these had pronounced symptoms in VAS-IBS parameters during treatment, but preserved HRQOL and no gastrointestinal symptoms at the 5-year follow-up. She had not been subjected to subsequent GnRH treatments. The other patient had few symptoms in VAS-IBS both before and during treatment, but was unfortunately lost to follow-up. Patients with antibodies against GnRH-R before treatment had more abdominal pain compared to controls at baseline (81 (56–93) and 93 (77–97), respectively, p = 0.047) and more influence by gastrointestinal symptoms on daily life (83 (40–94) and 94 (79–98), respectively, p = 0.020).

**Table 2:** The prevalence of antibodies against gonadotropin-releasing hormone (GnRH), luteinizing hormone (LH), and their receptors

	**Patients before IVF**	**Patients after IVF**	**Blood donors**	**BD vs. before IVF**	**Before vs. after IVF**
	**N = 96**	**N = 96**	**N = 169**	**P-value**	**P-value**
GnRH IgM, n (%)	5 (5.2)	3 (3.1)	4 (2.4)	0.292	0.721
GnRH-R IgM, n (%)	13 (13.5)	10 (10.4)	4 (2.4)	0.001	0.657
LH IgM, n (%)	5 (5.3)	2 (2.1)	5 (3.0)	0.504	0.444
LH IgG, n (%)	14 (14.6)	5 (5.3)	5 (3.0)	0.001	0.051
LH-R IgM, n (%)	6 (6.3)	6 (6.3)	6 (3.6)	0.362	1.000
LH-R IgG, n (%)	9 (9.4)	9 (9.4)	4 (2.4)	0.016	1.000

BD = Blood donors, IVF = in vitro fertilization, n (%) = number of patients. Antibody levels above the cut-off of relative units >97.5^th^ percentile in a cohort of 169 healthy female blood donors were considered as presence of antibodies. No IgG antibodies against GnRH or its receptor were found in patients. Fisher’s exact test (2-sided). P < 0.05 was considered statistically significant.

**Table 3:** The titer of antibody levels against gonadotropin-releasing hormone (GnRH), luteinizing hormone (LH), and their receptors

	**Patients before IVF**	**Patients after IVF**	**Blood donors**	**BD vs. before IVF**	**Before vs. after IVF**
	**RU**	**RU**	**RU**	**P-value**	**P-value**
GnRH IgM (≥85)	113 (102–136)	113 (89-)	159 (116–196)	0.190	0.607
GnRH-R IgM (≥75)	103 (88–256)	179 (102–350)	116 (86–134)	0.785	0.343
LH IgM (≥0.82)	0.9 (0.9-1.6)	1.1 (0.8-)	0.9 (0.8-2.2)	1.000	0.857
LH IgG (≥2.0)	3.0 (2.3-4.6)	4.3 (2.4-9.2)	2.7 (2.1-7.6)	0.839	0.511
LH-R IgM (≥166)	240 (194–504)	214 (185–649)	232 (158–310)	0.699	0.732
LH-R IgG (≥338)	452 (379–582)	406 (366–609)	559 (447–742)	0.330	0.863

BD = Blood donor, IVF = in vitro fertilization. Antibody levels are expressed as relative units (RU). The cut-off of RU >97.5^th^ percentile in a cohort of 169 healthy female blood donors were considered as presence of antibodies, and level was calculated only in positive samples. No IgG antibodies against GnRH or its receptor were found in patients. Values are given as median, interquartile range. Wilcoxon’s signed rank test or Mann-Whitney U-test were used. P < 0.05 was considered statistically significant.

The prevalence of LH- and LH-R IgG antibodies was significantly higher in patients before IVF treatment compared to controls, with a tendency to higher prevalence also compared to patients after treatment (Table 2). The antibody titers are shown in Table 3. Competitive ELISA regarding LH showed that the antibody-binding capacity to the solid phase was reduced with 43%–87%, dependent on the amount of antigen added (1.0–5.0 ng/ml) to the diluted serum before application on the microtiter plate.

## Discussion

Prospective evaluation of 124 patients who underwent IVF treatment showed that buserelin led to several gastrointestinal symptoms when administered. At 5-year follow-up, none of the 62 re-investigated patients (49%) had developed severe gastrointestinal motility disorder. The group had increased abdominal pain compared with prior treatment, and nine of the 62 women (15%) had pronounced symptoms that were interpreted as exacerbation or development of IBS. On the other hand, the psychological well-being had improved. Patients had higher prevalence of IgM GnRH-R antibodies and IgG LH and LH-R antibodies compared to controls, but without detectable antibody development during treatment.

Treatment with buserelin is associated with gastrointestinal side effects [19], and IVF is associated with many psychological aspects that also might affect gastrointestinal function [20]. The absence of antibody formation during treatment suggests that the previous described antibody occurrence in women with GnRH-induced dysmotility was secondary to the disease, and not due to the treatment *per se*[7,11]. Additional factors such as a previous susceptibility, genetic factors or concurrent infection might have been involved in these cases, explaining their GnRH-related severe dysmotility [7]. The fact that buserelin can lead to constipation and nausea and vomiting during treatment, underlines prior speculations regarding the role of GnRH on the ENS and gastrointestinal motor control [4,21]. The symptoms most prominent in this study during GnRH treatment are the same symptoms which have been most prominent in the patients with persistent dysmotility after IVF [7,11]. The persistent increase of abdominal pain may reflect a permanent damage to the ENS, in line with the enteric neurodegeneration evoked in a rat model [12]. In vitro fertilization renders a great psychological stress, and impaired psychological well-being just prior to treatment is not surprising [20]. After five years, infertile couples may either have got children or accepted the infertility, explaining the improved well-being at follow-up.

The specific role of GnRH in the gut is not completely elucidated, but GnRH analogs have been shown to inhibit gastric secretion and gastrin release in rat and dog [22,23], to inhibit cell proliferation in gastric epithelium [24], and to protect enteric rat neurons in culture when continuously stimulated [25], whereas shorter stimulation inhibits cell proliferation in gastric smooth muscle cells [26]. Furthermore, GnRH induces apoptosis and inhibited cell proliferation in several cancer cells [27,28] and induced enteric neurodegeneration in rat [12]. Two main explanations to the gastrointestinal effects evoked could be speculated on. First, endogenous GnRH is secreted into the hypothalamic portal circulation in a pulsatile fashion. It is rapidly degraded and barely detectable in peripheral circulation. In contrast, systemically administered GnRH analogs have longer half-life and cause greater exposure of the peripheral tissues [4,29]. GnRH and its receptors have been reported to be present and influence the rat digestive tract [5,23,24,26,30], and GnRH analogs have been suggested to act directly on enteric neurons through a complex combination of pathways and factors [21,29].

Second, or maybe concurrently, GnRH might act through the sex hormone axis as it initially raises circulating levels of FSH and LH [4]. Prolonged administration turns off the release of FSH and LH, the effect sought in the IVF setting [4]. It is possible that GnRH exerts its effect on the gastrointestinal tract indirectly; through pituitary LH release or absence. Luteinizing hormone has been shown to influence motor activity in rat small intestine [31]. Furthermore, LH-R were recently described on human enteric neurons [32], and the expression was decreased after repeated buserelin treatment in rats [12]. The positive effects of continuous treatment with GnRH analogs to patients with IBS may be due to the down-regulation of gonadotropins and gonadal sex hormones [9,10], as these hormones have negative effects on the gastrointestinal motility [31,33].

This is to our knowledge the first time as antibodies against LH-R have been analyzed in human serum. The patients expressed LH- and LH-R IgG antibodies and GnRH-R IgM antibodies to a greater extent than controls, already before IVF treatment. Infertility is common and affects over 10% of reproductive women [34]. Diagnostic categories of reasons for female infertility may involve endometriosis, tubal factors, uterine factors, ovarian factors, unexplained infertility, and multiple other causes [34,35]. Autoantibodies directed towards FSH, LH, and ovarial factors have been described in infertile women and could be indicative of an autoimmune disorder targeting for instance the ovary [35-37]. Thus, the current findings of increased autoantibodies already before treatment could be related to the infertility *per se* and reflect an increased autoimmune activity. The decreased prevalence of autoantibodies immediately after buserelin treatment may reflect a down-regulation of the immune system and IgG levels, described previously [38]. The association between gastrointestinal symptoms and antibody expression found in the present study has to be further studied in the future. However, the expression of LH-R in both genital organs and gastrointestinal tract [12,29,32], down-regulation of LH-R after buserelin treatment [12], and occurrence of serum antibodies against LH and LH-R, suggest that LH and its receptor may be one plausible explanation to the connection between gut and genital organs observed in women [2,3,7,21]. Antibodies against FSH or its receptor were not analyzed as these peptides have not been detected in the digestive tract [39].

The current study has several limitations, where the most apparent is the loss of patients to follow-up, with a response rate of 49% after one reminder. Patients who have experienced side effects of the treatment could theoretically be more prone to return the questionnaire. On the contrary, the patients who have got children after IVF are maybe not prone to report aggravated symptoms after the treatment. It would also have been preferable to have recruited the control group at the time of inclusion of patients and to have a 5-year follow-up also in the control group, since it is known that gastrointestinal symptoms in a Swedish population are varying over time [40]. However, in both the case of antibodies and symptoms, patients provide their own controls and the population-based material are merely intended to provide a benchmark control group, since IVF patients could represent a selected group. At inclusion, patients had more nausea and vomiting compared with controls. This may depend on that 60% of patients already had undergone IVF, and thus GnRH treatment, prior to this study, or to the many psychological factors associated with IVF [20]. Also, some of the 15 patients with past or concurrent endometriosis could have received GnRH analogs as treatment of their endometriosis, although GnRH analogs are not the first choice of treatment in this entity. To include patients for symptom registration, and not only blood samples, prior to the first treatment had been the most optimal.

## Conclusions

We can conclude that although many patients experience gastrointestinal side effects during treatment with the GnRH analog buserelin, and an increase in abdominal pain and improved psychological well-being persists after five years, no severe dysmotility cases were prospectively observed amongst 62 patients in a 5-year perspective. Neither was there any detected development of antibodies against buserelin, GnRH, LH or their receptors related to buserelin treatment. The results are in line with prior large epidemiological studies stating that IVF treatment seems reasonably safe [41]. However, it remains to identify which patients who are at risk to develop serious, persistent gastrointestinal side effects after GnRH treatment [7,11].

## Abbreviations

FSH: Follicle-stimulating hormone; GnRH: Gonadotropin-releasing hormone; hCG: Human chorionic gonadotropin; IVF: In vitro fertilization; LH: Luteinizing hormone; SF-36: 36-item Short-Form questionnaire; VAS-IBS: Visual Analogue Scale for Irritable Bowel Syndrome.

## Competing interests

The authors declare that they have no competing interests.

## Authors’ contributions

All authors together designed the study. OH and BO collected the data. BR developed and ran the ELISA tests. MB has developed the VAS-IBS. OH and BO performed the statistical calculations and wrote the manuscript. BO financially supported the study. All authors contributed to the manuscript with constructive criticism, and read and approved the final manuscript.

## Pre-publication history

The pre-publication history for this paper can be accessed here:

http://www.biomedcentral.com/1471-2393/13/201/prepub

## Supplementary Material

Additional file 1: Table S1 Concurrent diseases in patients subjected to in vitro fertilization. Click here for file
