# Supplementary material for: Autoantibodies and gastrointestinal symptoms in infertile women in relation to in vitro fertilization
Source: BMC Pregnancy Childbirth. 2013 Nov 5;13:201. doi: 10.1186/1471-2393-13-201 (PMC3826525; doi:10.1186/1471-2393-13-201)
Supplement: Additional file 1: Table S1 — Concurrent diseases in patients subjected to in vitro fertilization. [file 1471-2393-13-201-S1.doc]

TABLE 1. Concurrent diseases in patients subjected to in vitro fertilization

| Diseases | Prevalence n (%) |
| --- | --- |
| No concurrent disease | 73 (59) |
| Amenorrhea | 1 (1) |
| Anovulation | 5 (4) |
| APC resistance | 3 (2) |
| Asthma | 8 (6) |
| Atonic bladder | 1 (1) |
| Crohn´s disease | 1 (1) |
| Depression | 3 (2) |
| Dyspepsia | 2 (2) |
| Endometriosis | 15 (12) |
| Fibromyalgia | 1 (1) |
| Hypothyroidism | 8 (6) |
| Lactose intolerance | 2 (2) |
| Obesity | 1 (1) |
| Pituitary adenoma | 1 (1) |
| Polycystic ovary syndrome | 3 (2) |
| Rheumatoid arthritis | 2 (2) |

Patients presented can have more than one disease. APC = Activated protein C.
